# Distribution of ncRNAs expression across hypothalamic-pituitary-gonadal axis in *Capra hircus*

**DOI:** 10.1186/s12864-018-4767-x

**Published:** 2018-05-30

**Authors:** Emanuele Capra, Barbara Lazzari, Stefano Frattini, Stefania Chessa, Beatrice Coizet, Andrea Talenti, Bianca Castiglioni, Paolo Ajmone Marsan, Paola Crepaldi, Giulio Pagnacco, John L. Williams, Alessandra Stella

**Affiliations:** 1 Istituto di Biologia e Biotecnologia Agraria, Consiglio Nazionale delle Ricerche, Lodi, Italy; 2 Parco Tecnologico Padano, Lodi, Italy; 3 Dipartimento di Medicina Veterinaria, Università degli studi di Milano, Milan, Italy; 4 Istituto di Zootecnica, Università Cattolica del Sacro Cuore, Piacenza, Italy; 5 Davies Research Centre, School of Animal and Veterinary Sciences, University of Adelaide, Roseworthy, Australia

**Keywords:** miRNA, HPG, Goat, Small-RNA, Reproduction

## Abstract

**Background:**

Molecular regulation of the hypothalamic-pituitary-gonadal (HPG) axis plays an essential role in the fine tuning of seasonal estrus in *Capra hircus*. Noncoding RNAs (ncRNAs) are emerging as key regulators in sexual development and mammalian reproduction. In order to identify ncRNAs and to assess their expression patterns, along the HPG axis, we sequenced ncRNA libraries from hypothalamus, pituitary and ovary of three goats.

**Results:**

Among the medium length noncoding RNAs (mncRNAs) identified, small nucleolar RNAs (snoRNAs) and transfer RNAs (tRNAs) were found to be more abundant in ovary and hypothalamus, respectively. The observed GC content was representative for different classes of ncRNAs, allowing the identification of a tRNA-derived RNA fragments (tRFs) subclass, which had a peak distribution around 32–38% GC content in the hypothalamus. Differences observed among organs confirmed the specificity of microRNA (miRNA) profiles for each organ system.

**Conclusions:**

Data on ncRNAs in organs constituting the HPG axis will contribute to understanding their role in the physiological regulation of reproduction in goats.

**Electronic supplementary material:**

The online version of this article (10.1186/s12864-018-4767-x) contains supplementary material, which is available to authorized users.

## Background

The hypothalamic-pituitary-gonadal (HPG) axis regulates reproduction in mammals from fetal development, through puberty to sexual maturity [1]. The coordination of peripheral organs with the central nervous system ensures that animal physiology is aligned with the external environment to optimize reproductive success [2]. The goat oestrous cycle is accompanied by hormonal changes along the HPG axis, that orchestrate morphological and physiological changes in the ovaries leading to ovulation and preparation of the reproductive tract for oocyte maturation, sperm transport, fertilization, and embryo implantation [3].

Noncoding RNAs (ncRNAs) are involved in a remarkable variety of biological functions. These RNAs are divided into several families based on their size and biogenesis pathways, and act as part of RNA-protein complexes in regulating gene expression [4]. Regulatory ncRNAs can be placed in three major classes based on transcript size: small (sncRNAs), medium (mncRNAs) and long noncoding RNAs (lncRNAs) [5]. These different ncRNAs are further classified based on sequence or structure conservation, subcellular localization and function, association with annotated protein-coding genes and other DNA elements of known function [6]. Various bioinformatic tools are available that use either sequence motifs or structural parameters to detect novel ncRNAs [7] from sequence data.

Various classes of ncRNAs have roles in promoting the mammalian sexual phenotype [8]. Antisense long non coding RNAs (lncRNAs) may affect the expression and function of genes regulating sex determination and gonad development, such as the forkhead box L2 (*FOXL2*) [9]. The U17 short nucleolar RNA (snoRNA) has been shown to regulate a small non-coding RNA produced from the *Snhg3* introns which influence cellular cholesterol trafficking in the ovary, and thus may play a role in regulating steroid hormone production and postnatal gonadal maturation [10]. The role of miRNAs in modulating the HPG axis is well documented. For example, in the hypothalamus Lin28/let-7 expression patterns are associated with the onset of puberty [11]. Lin28a and Lin28b expression decreases during the onset of puberty transition and Lin28b expression in the ovary may be affected by environmental cues to delay puberty and/or follicular development [12]. MiR-361-3p is involved in regulating FSH secretion in a pig pituitary cell model [13]. Examination of genome-wide miRNA expression in goats suggested that miR-424-5p and miR-29a regulate muscle development [14] and that miRNAs play an important role in endometrial receptivity [15]. It was also observed that miRNA differ in their expression in the three stages of hair follicle cycles in cashmere goats [16], as well as in the ovary between pregnant and non-pregnant individuals, with 294 miRNA upregulated and 113 downregulated in pregnant goats [17].

Although miRNAs and other ncRNAs have shown to play a role in the regulation of tissue development and function, little is known about their tissue specification that to organ functionality along the reproductive axis.

In the present study, the ncRNA repertoires of hypothalamus, pituitary and ovary were investigated in *Capra hircus* by use of NGS.

## Methods

### Animals and tissue collection

Three adult female Saanen goats, aged 43.3 ± 3.2 months (mean ± SD) and weighing 55.0 ± 2.3 kg (mean ± SD), reared in the same group and on the same farm were sacrificed at the end of their productive life. The private owner agreed to yield them to the present research instead of the slaughterhouse with full consciousness about the purpose of the Project.

The experimental design was approved by the Animal Ethic Committee of the University of Milan. Animals were transported, anesthetized (Ketamine, 5 mg/kg/IV and Diazepan 1 mg/kg/IV), sacrificed by receiving a single intravenous (IV) bolus injection of a 10 mL solution of embutramide, mebezonium iodide and tetracaine hydrochloride (Tanax) and organs were collected according to the European Directive 2010/63/EU on the protection of animals used for scientific purposes. Samples of the hypothalamus, pituitary and ovaries were collected from each goat. The samples were immediately frozen in liquid nitrogen and ground to fine powder using mortar and pestle and stored at − 80 °C until RNA extraction.

### RNA isolation

Total RNA was isolated from each sample using Trizol (Invitrogen, Carlsbad, CA) and purified by the NucleoSpin® miRNA kit (Macherey-Nagel, Germany), following the protocol recommended by the manufacturer to prepare small and large RNA in one fraction (total RNA). RNA concentration and quality was determined using an Agilent 2100 Bioanalyzer (Santa Clara, CA). The isolated RNA was stored at − 80 °C.

### Library preparation and sequencing

Small noncoding RNA (sncRNA) libraries were generated using the TruSeq Small RNA Library Preparation kit according to manufacturer’s instructions (Illumina). The libraries were then pooled (3 goats for 3 organs) and purified on a Pippin Prep system (Sage Science, MA, USA) to recover two fractions of 125 to 167 nt (fraction 1, Illumina adapters included), containing mature miRNAs, and of 168 to 300 nt (fraction 2, Illumina adapters included), containing other ncRNAs, respectively. The quality and yield after sample preparation was measured with an Agilent 2200 Tape Station, High Sensitivity D1000 (Santa Clara, CA). Libraries were quantified by Real Time PCR with KAPA Library Quantification Kits (Kapa Biosystems, Inc. MA, United States). Libraries from fraction 1 and fraction 2 were sequenced on Miseq desktop sequencer (Illumina) with 50-base and 150-base single reads, respectively.

### Bioinformatic analysis

All sequences were quality checked with FastQC (http://www.bioinformatics.babraham.ac.uk/projects/fastqc/) and trimmed with Trimmomatic (minimum sequence quality 30 and minimum sequence length 14) [18].

#### ncRNA

ncRNA sequences from fraction 2 from hypothalamus, pituitary and ovary were collected and separately collapsed in silico into three non-redundant datasets with the Fastx-Toolkit collapser tool (http://hannonlab.cshl.edu/fastx_toolkit/). These datasets were compared by BLASTn to the RNAcentral database sequences [19] and hits having at least 90% coverage and 90% similarity to the RNAcentral entries were assigned to the RNA class of the corresponding RNAcentral sequence. Statistical analyses were performed with EdgeR [20] and the GLM model was applied to identify sequences with differential expression among the three datasets. Differential expression analyses across the three organs were run with the Bioconductor edgeR package (GLM model, FDR < 0.01 and LogFC> 1.5).

GC content of goat ncRNA sequences and entries present in the small human noncoding RNAs DASHR database (*http://lisanwanglab.org/DASHR/smdb.php#tabData*) [21], were calculated with Geecee (http://www.bioinformatics.nl/cgi-bin/emboss/geecee).

#### miRNA data analysis

miRNA detection and discovery were carried out with Mirdeep2 on Illumina high quality trimmed sequences from fraction 1. *Capra hircus* miRNA sequences available at MirBase (http://www.mirbase.org/) were used to identify known miRNA in the trimmed sequences. Known miRNA from related species (sheep, cow and horse) available at MirBase were also used by Mirdeep2 to support the identification of novel goat miRNAs. The Mirdeep2 quantifier module was used to quantify expression and retrieve counts for the known and novel miRNAs. Differential expression analyses across the three organs were run with the Bioconductor edgeR package (FDR < 0.01 and LogFC> 1.5). MiRNA cluster analysis was performed with the Genesis software to identify and visualize patterns within the datasets [22]. MiRNA target prediction was performed by Ingenuity Pathway Analysis (IPA, Ingenuity System, www.ingenuity.com). Human homologous miRNAs were analyzed with.

microRNA Target filter (IPA) to attribute (experimentally observed) target genes. Gene ontology (GO) classification of miRNA target mRNA was performed according to classical GO categories, using the Cytoscape plug-in ClueGO which integrates GO [23] and enhances biological interpretation of large lists of genes.

### miRNA validationby real time PCR qRT-PCR

RNA samples isolated from each organ were retro-transcribed with miScript II RT Kit following manufacturer’s instructions (Qiagen, Inc., Valencia, CA USA). Quantitative Real Time PCR (RT-PCR) was carried out on cDNAs with 7900HT Fast Real-Time PCR System (Applied Biosystems, Carlsbad, California, USA). Reactions were carried out in 10 μl volumes containing 1 M of each primer, 2 μl cDNA (see above), and 5 μl 2× Power SYBR® Green PCR Master Mix (Applied Biosystems) according to manufacturer protocols. The primers used for chi-miR-141, chi-miR-7, chi-miR-9-5p and chi-miR-10a-5p quantification, were designed using miRprimer software [24], (Additional file 1). For miR-124a-1 quantification, the bta-mir-124a-1 miScript Primer Assay (Qiagen, Inc., Valencia, CA USA) was used. Normalization used the small nucleolar snoRNA as reference, C/D Box 95 SNORD95 miScript Primer (Qiagen, Inc., Valencia, CA USA). Negative controls using water in place of samples were performed alongside each reaction. Reactions were run using the cycling parameters of 95 °C for 10 min, plus 40 cycles of 95 °C for 15 s, and 60 °C for 1 min. Relative expression levels and significance for each treatment were calculated separately using the 2-Ct method [25].

## Results

### Libraries preparation

**Fig. 1:**
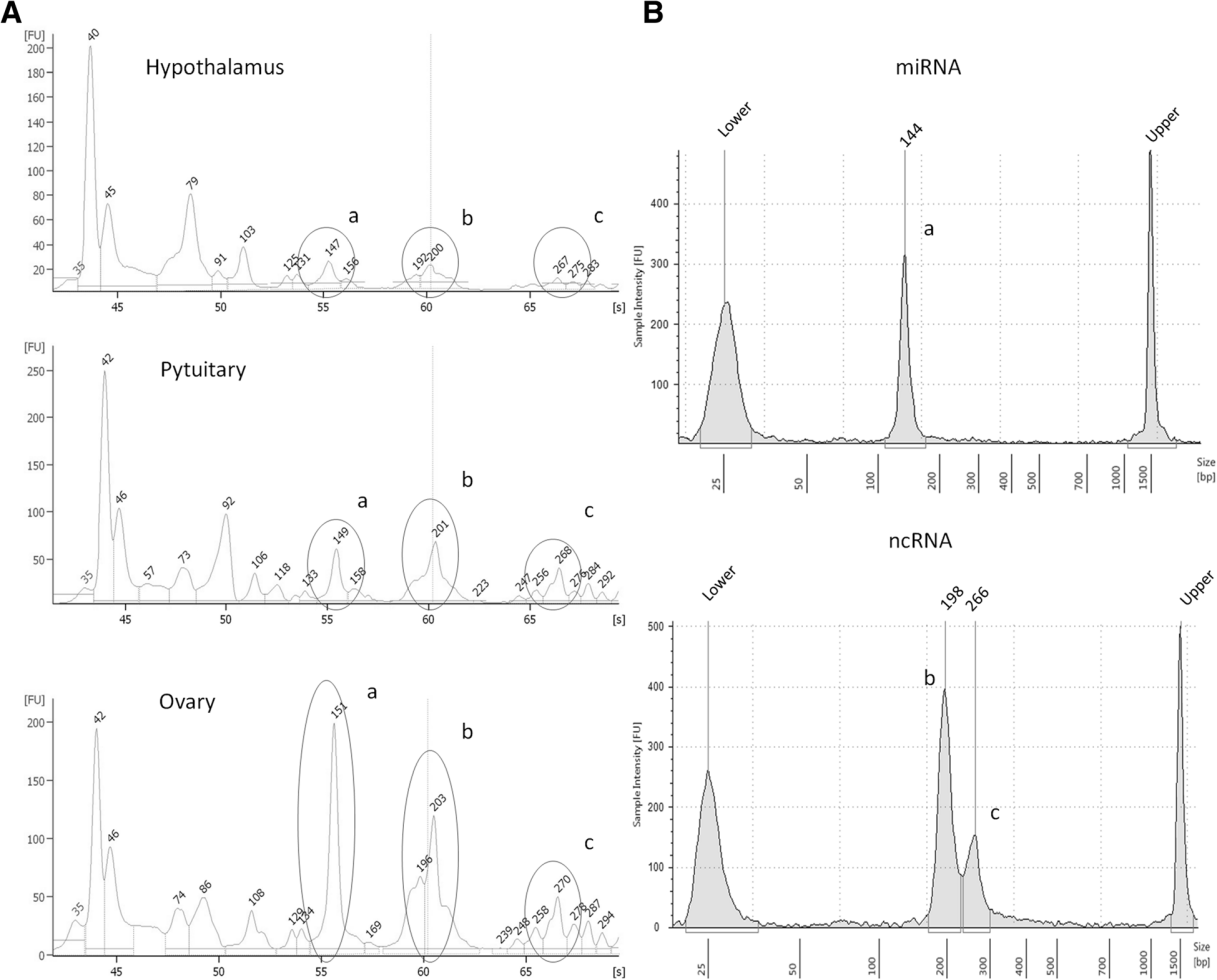
Small RNA libraries preparation. **A** Agilent 2100 bioanalyzer profile of a small RNA library obtained from RNA extracted from Hypothalamus, Pituitary and Ovary. **B** Agilent Tape station profile of a small RNA library fraction obtained by size-selection with pippinprep: miRNA libraries (144 bp), ncRNA libraries (198 and 266 bp). In circle sRNA libraries isolated in fraction 1 and fraction 2: **a**) 144 bp, **b**) 198 bp and (**c**) 266 bp. Illumina adapters were120 bp long

NcRNA libraries obtained from hypothalamus, pituitary and ovaries have three major size peaks, corresponding to 149, 201 and 268 bp (Fig. 1a). To represent the variety of small RNAs in goat, two small RNA fractions from pooled libraries (3 tissues for 3 animals) were selected: i) “fraction 1” containing miRNAs (about 20–30 nt in length) and ii) “fraction 2” containing other ncRNAs (70–140 nt); Illumina adapters were about 120 bp in size (Fig. 1b). Both fractions were sequenced and analyzed separately for their miRNAs and ncRNAs content. Data are available in the Sequence Reads Archive (SRA), BioProject accession number, SRP136431.

### ncRNA analysis

**Fig. 2:**
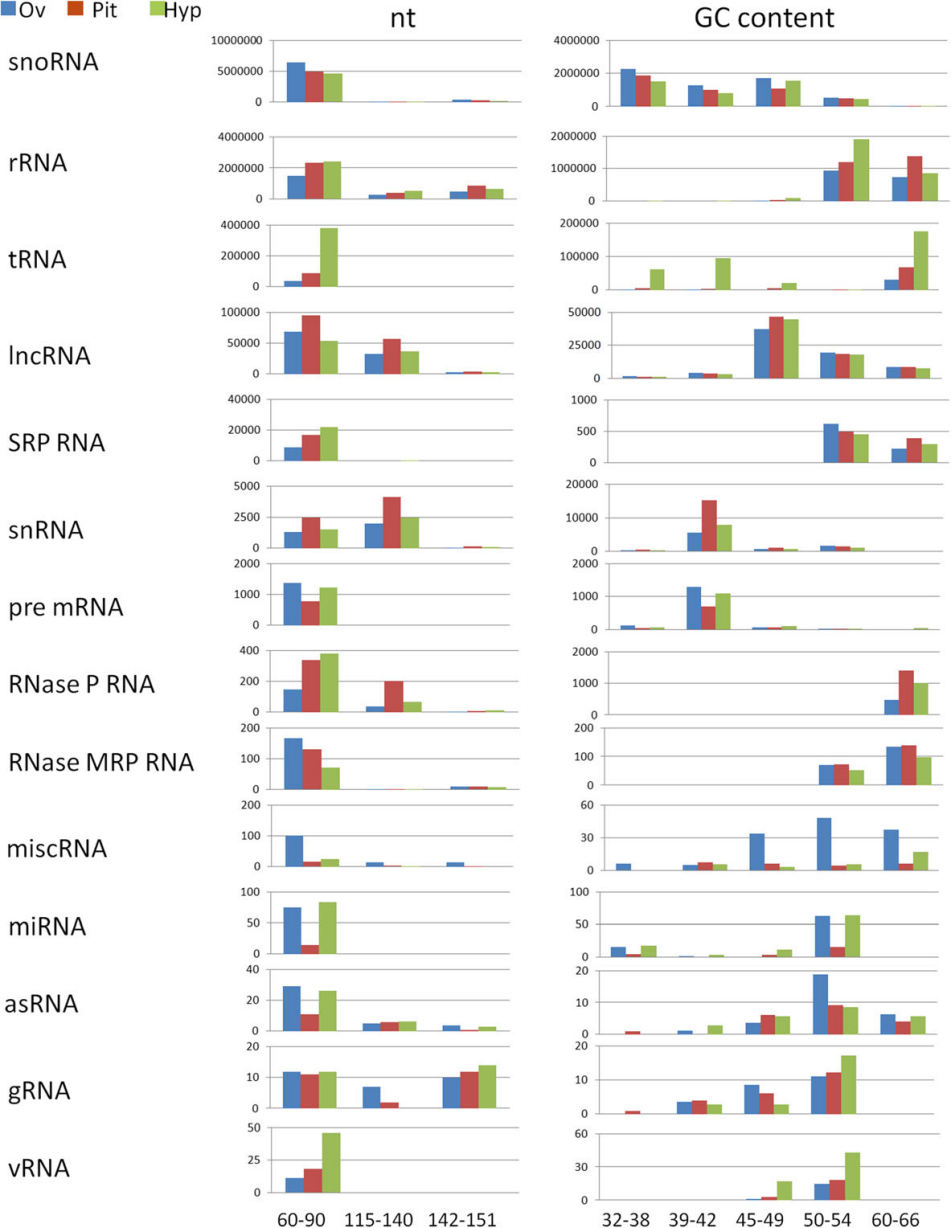
Distribution of different ncRNA classes in function of nucleotide length (nt) and GC content percentage (GC content) for the reads mapped against the RNAcentral database sequence collection. For each category the relative aboundance in each organis was reported (Y axis)

Miseq sequencing of fraction 2 (ncRNA 70-140 nt) resulted in 22,309,383 total of raw reads, with an average of 2,447,141 reads per sample. FastQC analysis grouped trimmed sequences depending on the nucleotide length and GC content (Additional file 2). In order to classify the sequenced *Capra hyrcus* ncRNAs according to the known ncRNA classes available in literature, homology searches against the sequence dataset of the RNAcentral non-coding RNA sequence database [19] were carried out (Fig. 2). A high percentage of reads was assigned to small nucleolar RNA (snoRNA), ribosomal RNA (rRNA), transfer RNA (tRNA), lncRNA and signal recognition particle (SRP RNA), while small nuclear (snRNA) and precursor RNA, ribonuclease P and MRP RNA (Rnase P RNA and RNAse MRP RNA), miscellaneous RNA (miscRNA), miRNA, antisense RNA (asRNA), guide RNA (gRNA) and vault RNA (vRNA) were less abundant. The ncRNA size and GC content distributions were consistent across all three organs. The majority of the ncRNAs were in the 60–90 nt size range. There was also a large number of lnRNAs, snRNAs and Rnase P RNAs reads ranging from 115 to 140 nt long. The distribution of gRNAs showed two peaks, corresponding to 60-90 nt and 142-151 nt. Different ncRNA classes were distributed according to GC content: sequences with low GC percentage were observed for snoRNA (peak at 32–38%), snRNA and precursor RNA (peak at 39–42%); a GC content near 50% was observed for lncRNA (peak at 45–49%) and rRNA, SRP RNA, miscRNA, miRNA, antisense RNA, guide RNA and vault RNA (peak at 50–54%); and a high GC content was observed for tRNA, RnaseP and Rnase MRP (peak at 60–66%). This highlights that in most cases ncRNA classes are characterized by a defined GC content.

**Fig. 3:**
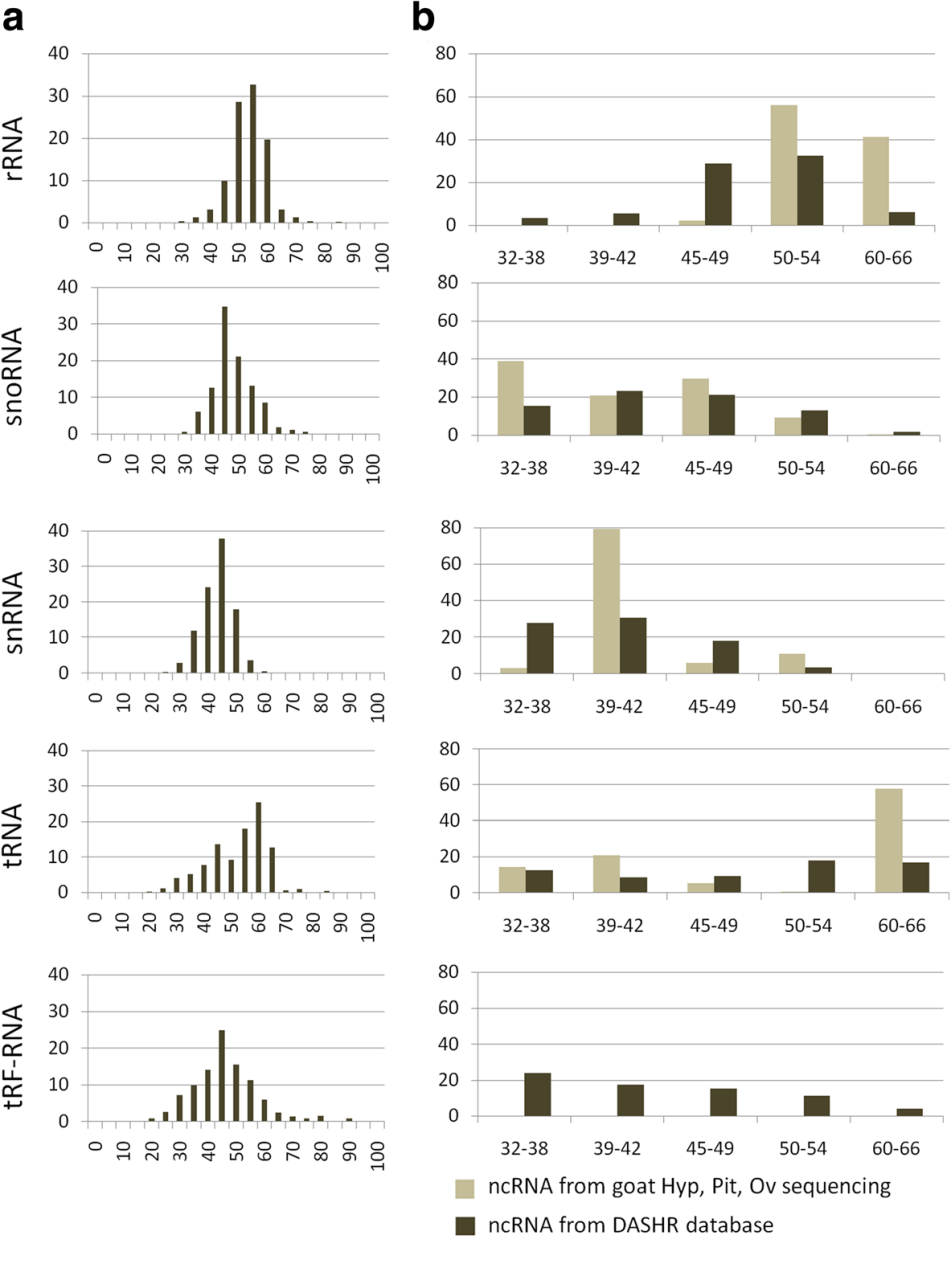
**a** total GC content distribution and (**b**) defined range GC content distribution, calculated with the EMBOSS geecee software on ncRNA entries from the small human noncoding RNAs DASHR database and on ncRNAs (experimentally observed data) for all three organs hypothalamus (Hyp), pituitary (Pit) and ovary (Ov) together in *Capra hircus*. rRNA, snoRNA, snRNA, tRNA were present in both DASHR database and experimental dataset. tRF-RNA class was present only in the DASHR database. On the Y axis percentage of the relative aboundance of each category of ncRNAs was reported. The X axis reports the percentage of GC content for each group

To validate this observation and further explore the intrinsic properties of ncRNAs, the GC content of entries present in the human sncRNAs DASHR database [19], including sequences belonging to different ncRNA classes (rRNA, snoRNA, snRNA, tRNA), was calculated. The distribution was similar to that observed for ncRNAs in *Capra hircus* (Fig. 3). However, ncRNA GC distribution between goat and human presented some differences, that could be probably related to the different dataset used, repository and sequencing data for human and goat respectively.

The DASHR database contains the tRNA-derived RNA fragments (tRFs), which had a peak at 32–38% GC content. A similar GC content was observed for the tRNA class in the hypothalamus that was probably associated with the presence of tRFs in this organ (compare Fig. 2 and Fig. 3). Expression levels of other ncRNA classes differed among organs, i.e. miscRNA, snoRNA and precursor RNA were more abundant in the ovary, lnRNA in the pituitary and tRNA and vRNA in the hypothalamus. MiRNA precursors were under-represented in the pituitary (Fig. 2). Statistical analysis, based on 1549 ncRNAs expressed in three organs, revealed that 8, 147 and 94 ncRNAs were differentially expressed between pituitary, hypothalamus and ovary (FDR < 0.01) (Additional file 3). The hypothalamus had a high proportion of tRNAs, whereas the ovary was enriched for snoRNAs (Additional file 4).

### miRNA analysis

**Fig. 4:**
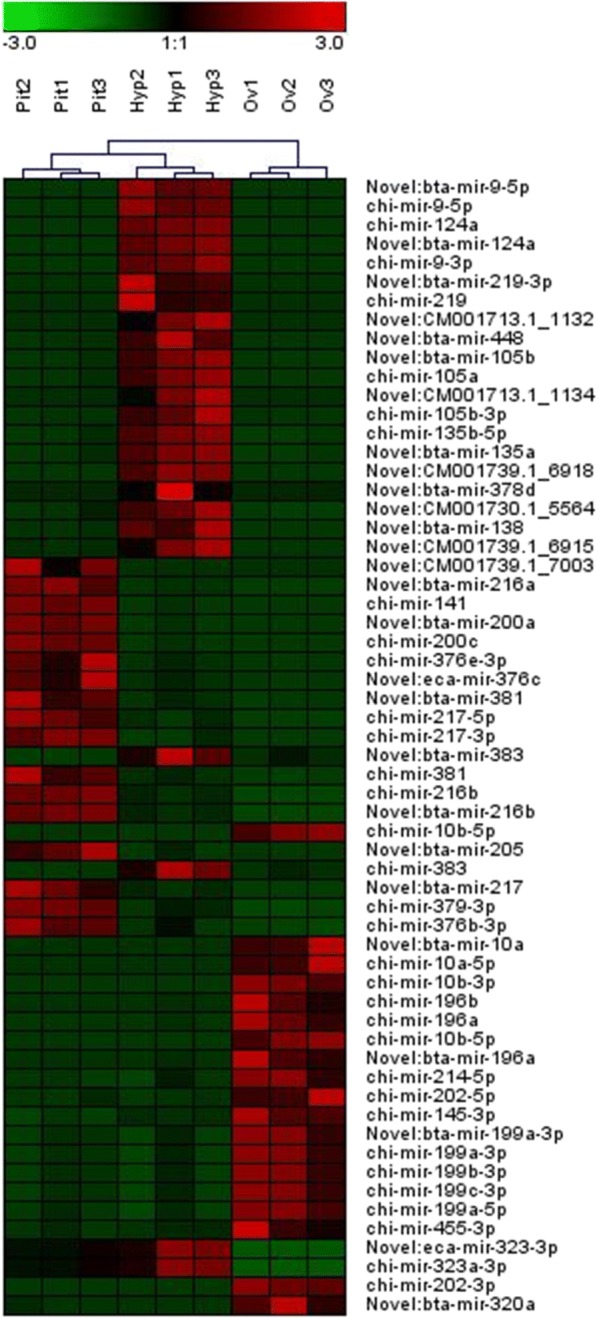
Hierarchical clustering obtained from normalized miRNA count for each replicate (1, 2, 3) in the three organs: hypothalamus (Hyp), pituitary (Pit) and ovary (Ov). A subset of miRNAs showing the highest variance among organs is reported. Red indicates an increase in expression and green a decrease in expression relative to the mean expression of 60 miRNAs

Miseq sequencing of fraction 1 (trimmed ncRNA of about 20-30 nt in length) resulted in 12,592,015 total raw reads, with an average of 1,399,112 reads per sample. The miRNA content in ncRNA libraries was explored by bioinformatic analysis of sequenced products using the miRDeep2 software. 785 known and putative miRNAs were identified and quantified in the tested samples. Among these miRNAs, 402 were already known in *Capra hircus* (chi-miRNAs), 222 had homology with known miRNAs from other species and 161 were predicted candidate novel miRNAs. After applying a stringent filtering (FDR < 0.01) for each target organ, 87, 70, and 233 miRNA were identified that were differentially expressed between pituitary, hypothalamus and ovary, respectively. The differential expression was obtained by comparing the expression in one organ versus the expression in the other two organs (Fig. 4). A list of organ-specific overexpressed miRNAs is given in Additional file 5.

Target genes of organ specific upregulated miRNAs found in this study were predicted, and related pathways identified. 6, 13 and 25 miRNA upregulated in pituitary, hypothalamus and ovary targeted 54, 329 and 970 experimental observed mRNA respectively. The canonical pathway analysis revealed that many genes were involved in fibroblast growth factor and epidermal growth factor response in the pituitary. Pathways related to the regulation of macromolecule metabolic process, organ development, cellular and developmental processes were prevalently targeted by the miRNA found upregulated in the hypothalamus and ovary (Additional file 6).

Differential expression of specific miRNAs in each organ was confirmed by qRT-PCR. MiR-141 and miR-7 were highly expressed in the pituitary and miR-9 and miR-124 highly expressed in the hypothalamus, whereas miR-10a-5p had the highest level of expression in the ovary (Additional file 7).

## Discussion

Goat mncRNA profiling of three organs according to GC content, showed five major peaks (GC content of peaks: 32–38%, 39–42%, 45–49%, 50–54%, and 60–66%), leading us to postulate that different mncRNAs have specific GC contents. Evaluation of distribution of GC content for different classes of small human noncoding RNAs available in the DASHR database supports our results. Although GC content was one of the most useful features for separating ncRNAs from other genomic elements [26, 27], we describe a deviation from random GC content for each mncRNA class.

The CG content is an important feature that affects function and stability of RNA: CG rich mRNA is more efficiently translated, affecting protein products levels [28]. GC composition also influences the degradation rate of mRNAs [29] and lncRNAs [30] and affects stability of RNA secondary structure [31]. It has been suggested that GC content around splice sites affects splicing through pre-mRNA secondary structures [32]. GC content has also been found to influence the function of sncRNA. Short interfering RNA (siRNA) GC-content correlates with RNA interference (RNAi) efficiency [33]. GC-content of synonymous codons in coding sequences is proven to have an impact on amino acid usage [34].

The relative abundance of the different mncRNA classes was similar in all three caprine organs: snoRNA, rRNA and tRNA were the most represented, in agreement with quantitative data on the expression landscape of small human noncoding RNA from other tissues available in the DASHR database.

In the current version of the database, the distribution of the various ncRNAs classes is different for different tissues. We found that hypothalamus, pituitary and ovary ncRNAs content is specific for each of the goat organs. The hypothalamus from goat was enriched for tRNAs and tRFs whereas ovary had an high level of snoRNAs. Intriguingly, goat hypothalamus expressed a high level of tRNA^Gly(GCC)^ and tRNA^Val(AAC)^. The same type of tRNA^Val^ and tRNA^Gly^ derived fragments were observed to be specifically produced in a controlled fashion in rat brain exposed to ischemia [35]. The level of tRFs was observed to increase when tRF targets decreased with age in rat brain [36]. tRNA-derived small RNAs served as novel signaling molecules in the response to stress [37, 38]. The high level of tRF found in goat hypothalamus may be important for maintaining a correct epigenetic asset and regulating organ function.

SnoRNAs regulate gene expression, playing a central role in ribosome biogenesis. However, many snoRNAs have not been ascribed a function, suggesting that they may have a different cell functionality [39]. Goat hypothalamus highly expresses SNORD109A, SNORD114 and SNORD116. Recently, an updated human snoRNAome based on snoRNAs from RFAM-based predictions, generated by the GENCODE consortium, found SNORD116 family and SNORD109 to be specifically overexpressed in neurons [40].

*Capra hircus* ovary was enriched in many snoRNA. The relative overexpression of SNORD (58, 93, 19, 69, 101, 46, 58, 121A, 58, 19b, 24, 38, 12 and 106) in goat ovary, matched snoRNAs profiling between hypothalamus and ovary from juvenile female sheep collected in Expression Atlas (http://www.ebi.ac.uk/gxa/home). On the contrary, three of the ovary overexpressed snoRNA in goat SNORD (18, 42 and 25) showed an opposite expression.

MiRNAs are regulators of gene expression that exhibit tissue and developmental-specific patterns and contribute in maintaining tissue homeostasis [41, 42]. In the present study, several specific miRNAs were predominantly expressed in one particular organ. MiR-141, miR200a and miR-7 were expressed in the pituitary gland while miR-124, miR-128 and miR-9 were highly expressed in the hypothalamus. This has also been observed in rodents [41] and in humans [43]. In the present study high levels of expression of miR-10b, miR-125b, miR-143, miR145, miR199b, miR21 and miR-99a were recorded in the ovary. A recent review identified that these miRNAs were highly expressed in mammalian ovary [44].

## Conclusions

In summary, this study described the goat (*Capra hircus)* ncRNA expression profiles in the three organs of the HPG axis. Comparison of these data with similar data from other species, when it becomes available, will provide insights into the role of different ncRNAs in the reproductive process. Finally, the ncRNA profiling may serve as a reference for further studies investigating the peculiarities of goat reproductive physiology, including seasonality in both sexes.

## Additional files

Additional file 1: Primer list and sequences used for Real Time validation experiment. (XLSX 9 kb)

Additional file 2: FastQC analysis result summary for ncRNA sequences after trimming process. For each organ an example of the reads distribution in function of sequence length and per sequence GC content was reported. (DOCX 217 kb)

Additional file 3: List of differentially expressed ncRNA (DE-ncRNA) (FDR < 0.01) for Pit (Pituitary vs other organs) Hyp (Hypothalamus vs other organs) and Ov (Ovary vs other organs). For each organ DE-ncRNAs, RNAcentral identification code (Id), logFC, FDR, Type on ncRNA and annotation are reported. (XLSX 28 kb)

Additional file 4: Distribution of over-expressed ncRNA (DE-ncRNA) (FDR < 0.01 and LogFC> 0) for Pit (Pituitary vs other organs) Hyp (Hypothalamus vs other organs) and Ov (Ovary vs other organs). For each organ DE-ncRNAs were sorted by categories: long non-coding RNAs (lncRNAs), miscellaneous RNA (misc_RNA), precursor_RNA, ribosomal RNA (rRNA), small nucleolar RNA (snoRNA), signal recognition particle RNA SRP_RNA, transfer RNA (tRNA). (DOCX 15 kb)

Additional file 5: List of organ specific overexpressed miRNA (DOCX 16 kb)

Additional file 6: Pathways identified for (experimentally observed) genes targeted by upregulated miRNA expressed in the pituitary, hypothalamus and ovary. (XLSX 148 kb)

Additional file 7: Comparison between A) RNA-Seq and B) Real-time PCR data, for 5 miRNAs (miR-141, miR-7-5p, miR-9-5p, miR-124a, miR-10a-5p), obtained from each organ: hypothalamus (Hyp), pituitary (Pit) and ovary (Ov) and three replicate (1, 2, 3). (DOCX 70 kb)

Additional File 8: Novel miRNA mature sequences in fasta format. Fasta headers report the absolute genomic start position of the sequence or the ID of the similar miRNA for novel miRNA detected by similarity to other species (cow, sheep or horse). (TXT 16 kb)

Additional File 9: Novel miRNA precursors sequences in fasta format. Fasta headers report the absolute genomic start position of the sequence or the ID of the similar miRNA for novel miRNA detected by similarity to other species (cow, sheep or horse). (TXT 30 kb)

Additional File 10: Non-redundant dataset of ncRNA from ovary, pituitary and hypothalamus in fasta format. (TXT 64614 kb)

## Abbreviations

asRNA: Antisense RNA
FDR: False Discovery Rate
gRNA: Guide RNA
HPG: Hypothamous-Pituitary-Gonadal
IPA: Ingenuity Pathway Analysis
lncRNA: Long non coding RNA
miRNA: Microrna
miscRNA: Miscellaneous RNA
mncRNA: Medium non coding RNA
ncRNA: Non coding RNA
Rnase P RNA: Ribonuclease P RNA
rRNA: Ribosomal RNA
siRNA: Short interfering RNA
sncRNA: Small non coding RNA
snoRNA: Small nucleolar RNA
SRA: Sequence Reads Archive
SRP RNA: Signal recognition particle RNA
tRNA: Transfer RNA
vRNA: Vault RNA

## References

[1] Thackray VG, Mellon PL, Coss D (2010). Hormones in synergy: regulation of the pituitary gonadotropin genes. Mol Cell Endocrinol.

[2] Christensen A, Bentley GE, Cabrera R, Ortega HH, Perfito N, Wu TJ, Micevych P (2012). Hormonal regulation of female reproduction. Horm Metab Res.

[3] Fatet A, Pellicer-Rubio MT, Leboeuf B (2011). Reproductive cycle of goats. Anim Reprod Sci.

[4] Cech TR, Steitz JA (2014). The noncoding RNA revolution-trashing old rules to forge new ones. Cell..

[5] Esteller M (2011). Non-coding RNAs in human disease. Nat Rev Genet..

[6] St Laurent G, Wahlestedt C, Kapranov P (2015). The landscape of long noncoding RNA classification. Trends Genet.

[7] Bao M, Cervantes Cervantes M, Zhong L, Wang JT (2012). Searching for non-coding RNAs in genomic sequences using ncRNA scout. Genomics Proteomics Bioinformatics.

[8] McFarlane L, Wilhelm D (2009). Non-coding RNAs in mammalian sexual development. Sexual Dev.

[9] Cocquet J, Pannetier MF, Veitia M, RA. (2005). Sense and antisense Foxl2 transcripts in mouse. Genomics.

[10] Jinn S, Brandis KA, Ren A, Chacko A, Dudley-Rucker N, Gale SE, Sidhu R, Fujiwara H, Jiang H, Olsen BN (2015). snoRNA U17 regulates cellular cholesterol trafficking. Cell Metab.

[11] Sangiao-Alvarellos S, Manfredi-Lozano M, Ruiz-Pino F, Navarro VM, Sánchez-Garrido MA, Leon S, Dieguez C, Cordido F, Matagne V, Dissen GA (2013). Changes in hypothalamic expression of the Lin28/let-7 system and related microRNAs during postnatal maturation and after experimental manipulations of puberty. Endocrinology.

[12] Grieco A, Rzeczkowska P, Alm C, Palmert MR (2013). Investigation of peripubertal expression of Lin28a and Lin28b in C57BL/6 female mice. Mol Cell Endocrinol.

[13] Ye RS, Xi QY, Qi Q, Cheng X, Chen T, Li H, Kallon S, Shu G, Wang SB, Jiang QY (2013). Differentially expressed miRNAs after GnRH treatment and their potential roles in FSH regulation in porcine anterior pituitary cell. PLoS One.

[14] Ling YH, Ding JP, Zhang XD, Wang LJ, Zhang YH, Li YS, Zhang ZJ, Zhang XR (2013). Characterization of microRNAs from goat (Capra hircus) by Solexa deep-sequencing technology. Genet Mol Res.

[15] Song Y, An X, Zhang L, Fu M, Peng J, Han P, Hou J, Zhou Z, Cao B (2015). Identification and profiling of microRNAs in goat endometrium during embryo implantation. PLoS One.

[16] Yuan C, Wang X, Geng R, He X, Qu L, Chen Y (2013). Discovery of cashmere goat (Capra hircus) microRNAs in skin and hair follicles by Solexa sequencing. BMC Genomics.

[17] Zhang XD, Zhang YH, Ling YH, Liu Y, Cao HG, Yin ZJ, Ding JP, Zhang XR (2013). Characterization and differential expression of microRNAs in the ovaries of pregnant and non pregnant goats (Capra hircus). BMC Genomics.

[18] Bolger AM, Lohse M, Usadel B (2014). Trimmomatic: a flexible trimmer for Illumina sequence data. Bioinformatics.

[19] Bateman A, Agrawal S, Birney E, Bruford EA, Bujnicki JM, Cochrane G, Cole JR, Dinger ME, Enright AJ, Gardner PP (2011). RNAcentral: a vision for an international database of RNA sequences. RNA.

[20] Robinson MD, DJ MC, Smyth GK (2010). edgeR: a Bioconductor package for differential expression analysis of digital gene expression data. Bioinformatics.

[21] Leung YY, Kuksa PP, Amlie-Wolf A, Valladares O, Ungar LH, Kannan S, Gregory BD, Wang LS (2016). DASHR: database of small human noncoding RNAs. Nucleic Acids Res.

[22] Sturn A, Quackenbush J, Trajanoski Z (2002). Genesis: cluster analysis of microarray data. Bioinformatics.

[23] Bindea G, Mlecnik B, Hackl H, Charoentong P, Tosolini M, Kirilovsky A, Fridman WH, Pagès F, Trajanoski Z, Galon J (2009). ClueGO: a Cytoscape plug-in to decipher functionally grouped gene ontology and pathway annotation networks. Bioinformatics.

[24] Busk PK (2014). A tool for design of primers for microRNA-specific quantitative RT-qPCR. BMC Bioinformatics..

[25] Livak KJ, Schmittgen TD (2001). Analysis of relative gene expression data using real-time quantitative PCR and the 2(−Delta Delta C(T)). Methods.

[26] Lu ZJ, Yip KY, Wang G, Shou C, Hillier LW, Khurana E, Agarwal A, Auerbach R, Rozowsky J, Cheng C (2011). Prediction and characterization of noncoding RNAs in C. Elegans by integrating conservation, secondary structure, and high-throughput sequencing and array data. Genome Res.

[27] Hu L, Di C, Kai M, Yang YC, Li Y, Qiu Y, Hu X, Yip KY, Zhang MQ, Lu ZJ (2015). A common set of distinct features that characterize noncoding RNAs across multiple species. Nucleic Acids Res.

[28] Robinson R (2006). More GC means more RNA. PLoS Biol.

[29] Gallego Romero I, Pai AA, Tung J, Gilad Y (2014). RNA-seq: impact of RNA degradation on transcript quantification. BMC Biol.

[30] Clark MB, Johnston RL, Inostroza-Ponta M, Fox AH, Fortinim E, Moscato P, Dinger ME, Mattick JS (2012). Genome-wide analysis of long noncoding RNA stability. Genome Res.

[31] Galtier N, Lobry JR (1997). Relationships between genomic G+C content, RNA secondary structures, and optimal growth temperature in prokaryotes. J Mol Evol.

[32] Zhang J, Kuo CC, Chen L (2011). GC content around splice sites affects splicing through pre-mRNA secondary structures. BMC Genomics.

[33] Chan CY, Carmack CS, Long DD, Maliyekkel A, Shao Y, Roninson IB, Ding Y (2009). A structural interpretation of the effect of GC-content on efficiency of RNA interference. BMC Bioinformatics.

[34] Li J, Zhou J, Wu Y, Yang S, Tian D (2015). GC-content of synonymous codons profoundly influences amino acid usage. G3 (Bethesda).

[35] Li Q, Hu B, Hu GW, Chen CY, Niu X, Liu J, Zhou SM, Zhang CQ, Wang Y, Deng ZF (2016). tRNA-derived small non-coding RNAs in response to ischemia inhibit angiogenesis. Sci Rep.

[36] Karaiskos S, Grigoriev A. Dynamics of tRNA fragments and their targets in aging mammalian brain. F1000Res. 2016;5. pii: ISCB Comm J-2758. 10.12688/f1000research.10116.1 PMC5224686 28105302

[37] Kumar P, Anaya J, Mudunuri SB, Dutta A (2014). Meta-analysis of tRNA derived RNA fragments reveals that they are evolutionarily conserved and associate with AGO proteins to recognize specific RNA targets. BMC Biol.

[38] Saikia M, Jobava R, Parisien M, Putnam A, Krokowski D, Gao XH, Guan BJ, Yuan Y, Jankowsky E, Feng Z (2014). Angiogenin-cleaved tRNA halves interact with cytochrome c, protecting cells from apoptosis during osmotic stress. Mol Cell Biol.

[39] Dupuis-Sandoval F, Poirier M, Scott MS (2015). The emerging landscape of small nucleolar RNAs in cell biology. Wiley Interdiscip Rev RNA.

[40] Jorjani H, Kehr S, Jedlinski DJ, Gumienny R, Hertel J, Stadler PF, Zavolan M, Gruber AR (2016). An updated human snoRNAome. Nucleic Acids Res.

[41] Landgraf P, Rusu M, Sheridan R, Sewer A, Iovino N, Aravin A, Pfeffer S, Rice A, Kamphorst AO, Landthaler M (2007). A mammalian microRNA expression atlas based on small RNA library sequencing. Cell.

[42] Sun K, Lai EC (2013). Adult-specific functions of animal microRNAs. Nat Rev Genet.

[43] Ludwig N, Leidinger P, Becker K, Backes C, Fehlmann T, Pallasch C, Rheinheimer S, Meder B, Stähler C, Meese E, Keller A (2016). Distribution of miRNA expression across human tissues. Nucleic Acids Res.

[44] Li Y, Fang Y, Liu Y, Yang X (2015). MicroRNAs in ovarian function and disorders. J Ovarian Res.

