# Supplementary material for: Distribution of ncRNAs expression across hypothalamic-pituitary-gonadal axis in Capra hircus
Source: BMC Genomics. 2018 May 30;19:417. doi: 10.1186/s12864-018-4767-x (PMC5977473; doi:10.1186/s12864-018-4767-x)

Additional File 2. FastQC analysis result summary for ncRNA sequences after trimming processing. For each organ an example of the reads distribution in function of sequence lenght and per sequence GC content was reported.

1. Sequence lenght distribution for:

Pituitary


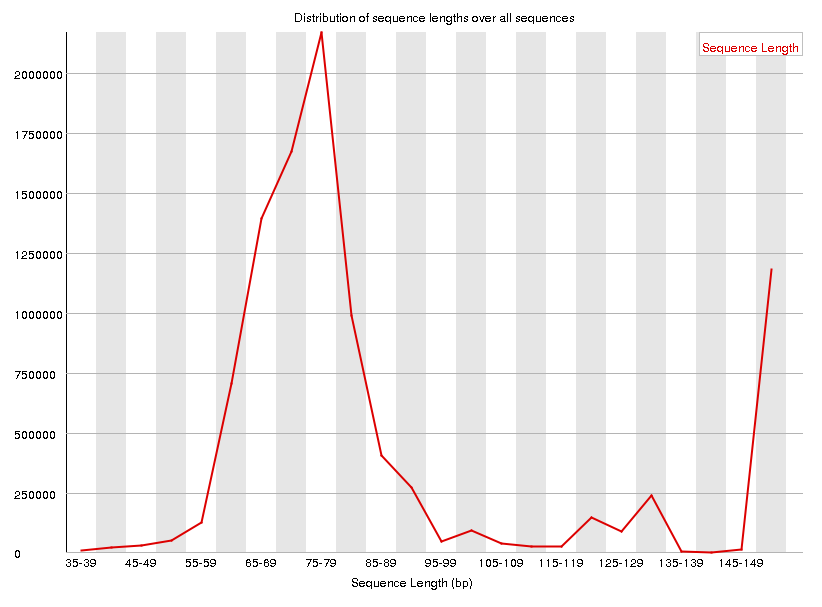


Ovary


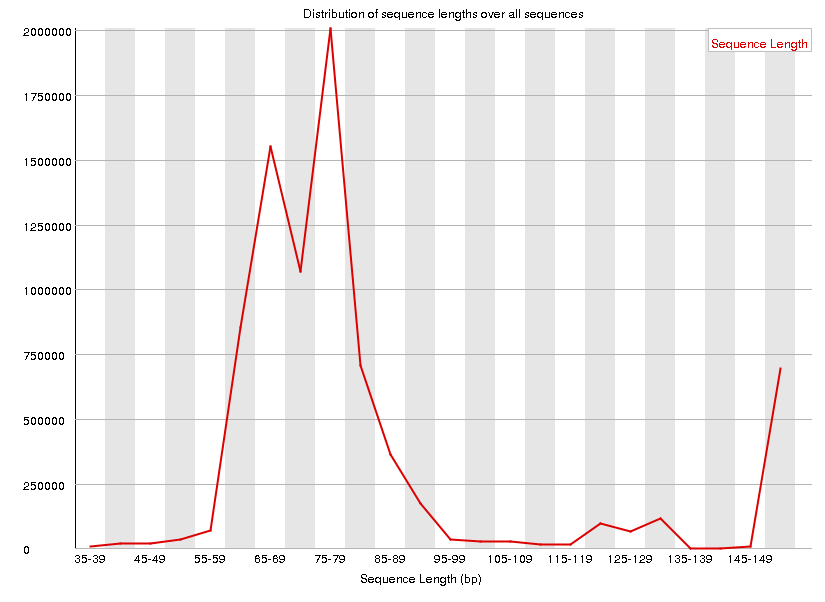


Hypothalamus


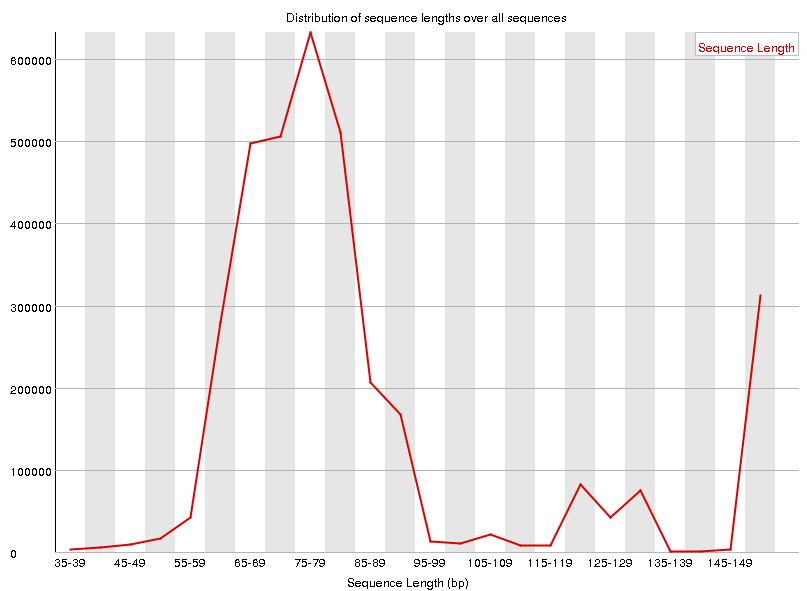


1. Per sequence GC content for:

Pituitary


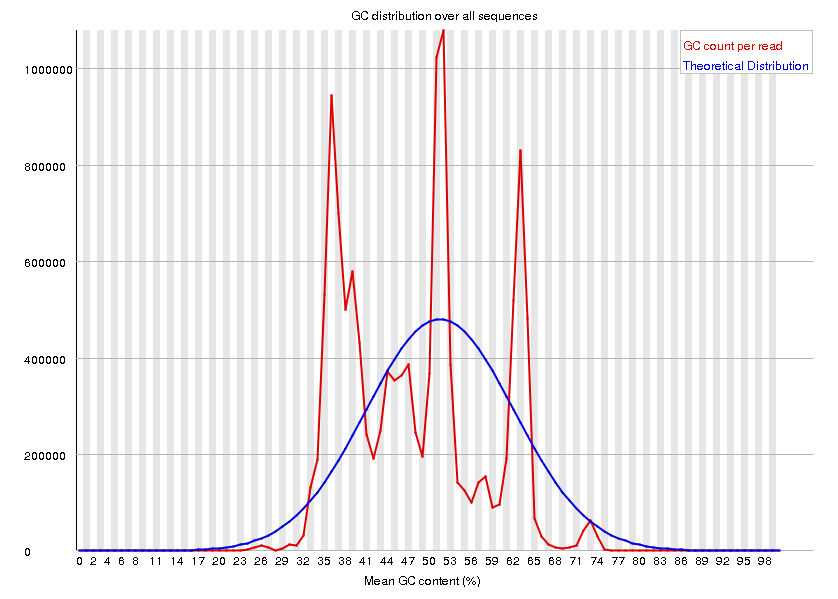


Ovary


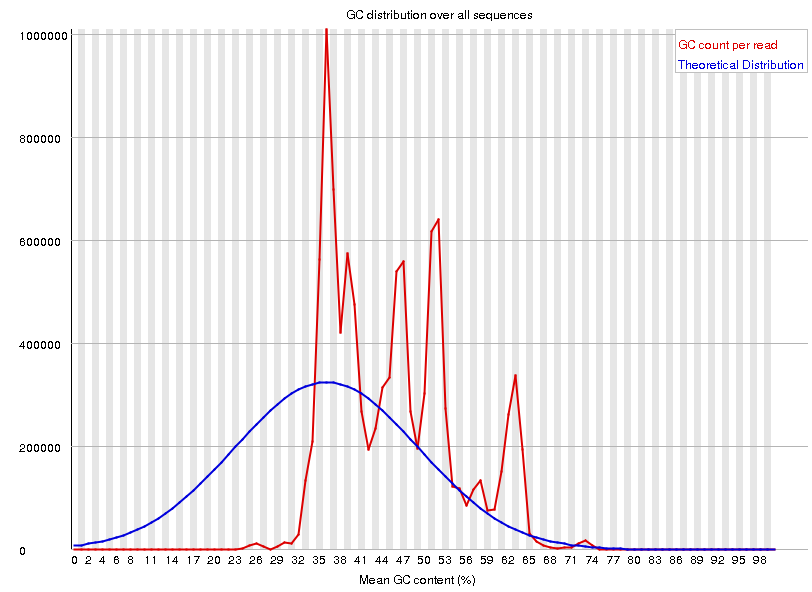


Hypothalamus


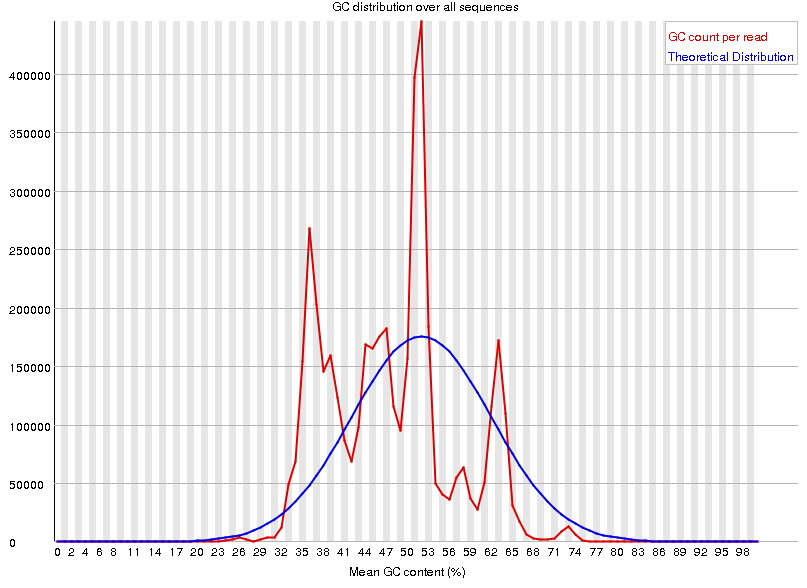

Supplement: Supplementary file 2 — FastQC analysis result summary for ncRNA sequences after trimming process. For each organ an example of the reads distribution in function of sequence length and per sequence GC content was reported. (DOCX 217 kb) [file 12864_2018_4767_MOESM2_ESM.docx]
