# Supplementary material for: Distribution of ncRNAs expression across hypothalamic-pituitary-gonadal axis in Capra hircus
Source: BMC Genomics. 2018 May 30;19:417. doi: 10.1186/s12864-018-4767-x (PMC5977473; doi:10.1186/s12864-018-4767-x)
Supplement: Supplementary file 4 — Distribution of over-expressed ncRNA (DE-ncRNA) (FDR < 0.01 and LogFC> 0) for Pit (Pituitary vs other organs) Hyp (Hypothalamus vs other organs) and Ov (Ovary vs other organs). For each organ DE-ncRNAs were sorted by categories: long non-coding RNAs (lncRNAs), miscellaneous RNA (misc_RNA), precursor_RNA, ribosomal RNA (rRNA), small nucleolar RNA (snoRNA), signal recognition particle RNA SRP_RNA, transfer RNA (tRNA). (DOCX 15 kb) [file 12864_2018_4767_MOESM4_ESM.docx]

Additinal File 4. Distribution of over-expressed ncRNA (DE-ncRNA) (FDR<0.01 and LogFC>0) for Pit (Pituitary vs other organs) Hyp (Hypothalamus vs other organs) and Ov (Ovary vs other organs). For each organ DE-ncRNAs were sorted by categories:long non-coding RNAs (lncRNAs), miscellaneous RNA (misc_RNA), precursor_RNA, ribosomal RNA (rRNA), small nucleolar RNA (snoRNA), signal recognition particle RNA SRP_RNA, transfer RNA (tRNA). In y axis the percentage of DE-ncRNA found for each classes was reported.
