# Supplementary material for: Distribution of ncRNAs expression across hypothalamic-pituitary-gonadal axis in Capra hircus
Source: BMC Genomics. 2018 May 30;19:417. doi: 10.1186/s12864-018-4767-x (PMC5977473; doi:10.1186/s12864-018-4767-x)
Supplement: Supplementary file 5 — List of organ specific overexpressed miRNA (DOCX 16 kb) [file 12864_2018_4767_MOESM5_ESM.docx]

Additional File 5. List of organ specific overexpressed miRNA

| **UP-Regulated Pituitary** | **UP-Regulated Hypothalamus** | **UP-Regulated Ovary** |
| --- | --- | --- |
| chi-let-7i-3p | chi-miR-105a | chi-let-7b-5p |
| chi-miR-136-5p | chi-miR-105b-3p | chi-let-7c-5p |
| chi-miR-141 | chi-miR-105b-5p | chi-miR-1 |
| chi-miR-186-3p | chi-miR-1249 | chi-miR-10a-3p |
| chi-miR-200a | chi-miR-124a | chi-miR-10a-5p |
| chi-miR-200c | chi-miR-128-3p | chi-miR-10b-3p |
| chi-miR-216b | chi-miR-135b-5p | chi-miR-10b-5p |
| chi-miR-217-3p | chi-miR-147-3p | chi-miR-125b-5p |
| chi-miR-217-5p | chi-miR-187 | chi-miR-130a-3p |
| chi-miR-376b-3p | chi-miR-20b | chi-miR-133a-3p |
| chi-miR-376b-5p | chi-miR-219 | chi-miR-143-3p |
| chi-miR-376c-3p | chi-miR-326-3p | chi-miR-143-5p |
| chi-miR-376c-5p | chi-miR-330-3p | chi-miR-145-3p |
| chi-miR-376e-3p | chi-miR-330-5p | chi-miR-145-5p |
| chi-miR-376e-5p | chi-miR-338-3p | chi-miR-146a |
| chi-miR-379-3p | chi-miR-340-3p | chi-miR-155-5p |
| chi-miR-379-5p | chi-miR-346-5p | chi-miR-195-3p |
| chi-miR-381 | chi-miR-363-3p | chi-miR-195-5p |
| chi-miR-3959-5p | chi-miR-485-3p | chi-miR-196a |
| chi-miR-411a-5p | chi-miR-592 | chi-miR-196b |
| chi-miR-429 | chi-miR-874-5p | chi-miR-199a-3p |
| chi-miR-494 | chi-miR-9-3p | chi-miR-199a-5p |
| chi-miR-582-5p | chi-miR-9-5p | chi-miR-199b-3p |
| chi-miR-7-5p | Novel:bta-miR-105b | chi-miR-199b-5p |
| Novel:bta-miR-200a | Novel:bta-miR-1249 | chi-miR-199c-3p |
| Novel:bta-miR-200c | Novel:bta-miR-124a | chi-miR-199c-5p |
| Novel:bta-miR-205 | Novel:bta-miR-128 | chi-miR-202-3p |
| Novel:bta-miR-216a | Novel:bta-miR-1298 | chi-miR-202-5p |
| Novel:bta-miR-216b | Novel:bta-miR-135a | chi-miR-21-3p |
| Novel:bta-miR-217 | Novel:bta-miR-138 | chi-miR-214-3p |
| Novel:bta-miR-299 | Novel:bta-miR-147 | chi-miR-214-5p |
| Novel:bta-miR-376b | Novel:bta-miR-187 | chi-miR-21-5p |
| Novel:bta-miR-379 | Novel:bta-miR-219-3p | chi-miR-224-5p |
| Novel:bta-miR-381 | Novel:bta-miR-326 | chi-miR-2284a |
| Novel:bta-miR-411a | Novel:bta-miR-338 | chi-miR-25-3p |
| Novel:bta-miR-494 | Novel:bta-miR-346 | chi-miR-28-3p |
| Novel:bta-miR-582 | Novel:bta-miR-370 | chi-miR-28-5p |
| Novel:bta-miR-7 | Novel:bta-miR-378d | chi-miR-320-3p |
| Novel:CM001726,1_4406 | Novel:bta-miR-448 | chi-miR-365-3p |
| Novel:CM001727,1_4754 | Novel:bta-miR-95 | chi-miR-449a-5p |
| Novel:CM001731,1_5621 | Novel:bta-miR-9-5p | chi-miR-449c |
| Novel:CM001739,1_7003 | Novel:CM001713,1_1132 | chi-miR-455-3p |
| Novel:eca-miR-376c | Novel:CM001713,1_1134 | chi-miR-455-5p |
| Novel:oar-miR-3959-5p | Novel:CM001717,1_2214 | chi-miR-483 |
|  | Novel:CM001727,1_4643 | chi-miR-497-5p |
|  | Novel:CM001728,1_4975 | chi-miR-92a-3p |
|  | Novel:CM001730,1_5564 | chi-miR-92b |
|  | Novel:CM001732,1_5836 | chi-miR-93-5p |
|  | Novel:CM001736,1_6537 | chi-miR-99a-3p |
|  | Novel:CM001739,1_6911 | chi-miR-99a-5p |
|  | Novel:CM001739,1_6915 | Novel:bta-miR-1 |
|  | Novel:CM001739,1_6918 | Novel:bta-miR-10a |
|  | Novel:eca-miR-3548 | Novel:bta-miR-125a |
|  | Novel:eca-miR-485-3p | Novel:bta-miR-143 |
|  | Novel:eca-miR-592 | Novel:bta-miR-145 |
|  |  | Novel:bta-miR-146b |
|  |  | Novel:bta-miR-151-5p |
|  |  | Novel:bta-miR-155 |
|  |  | Novel:bta-miR-196a |
|  |  | Novel:bta-miR-199a-3p |
|  |  | Novel:bta-miR-21-5p |
|  |  | Novel:bta-miR-224 |
|  |  | Novel:bta-miR-2299-3p |
|  |  | Novel:bta-miR-31 |
|  |  | Novel:bta-miR-320a |
|  |  | Novel:bta-miR-365-3p |
|  |  | Novel:bta-miR-377 |
|  |  | Novel:CM001738,1_6736 |
|  |  | Novel:eca-miR-28-3p |
