# Supplementary material for: Distribution of ncRNAs expression across hypothalamic-pituitary-gonadal axis in Capra hircus
Source: BMC Genomics. 2018 May 30;19:417. doi: 10.1186/s12864-018-4767-x (PMC5977473; doi:10.1186/s12864-018-4767-x)
Supplement: Supplementary file 7 — Comparison between A) RNA-Seq and B) Real-time PCR data, for 5 miRNAs (miR-141, miR-7-5p, miR-9-5p, miR-124a, miR-10a-5p), obtained from each organ: hypothalamus (Hyp), pituitary (Pit) and ovary (Ov) and three replicate (1, 2, 3). (DOCX 70 kb) [file 12864_2018_4767_MOESM7_ESM.docx]

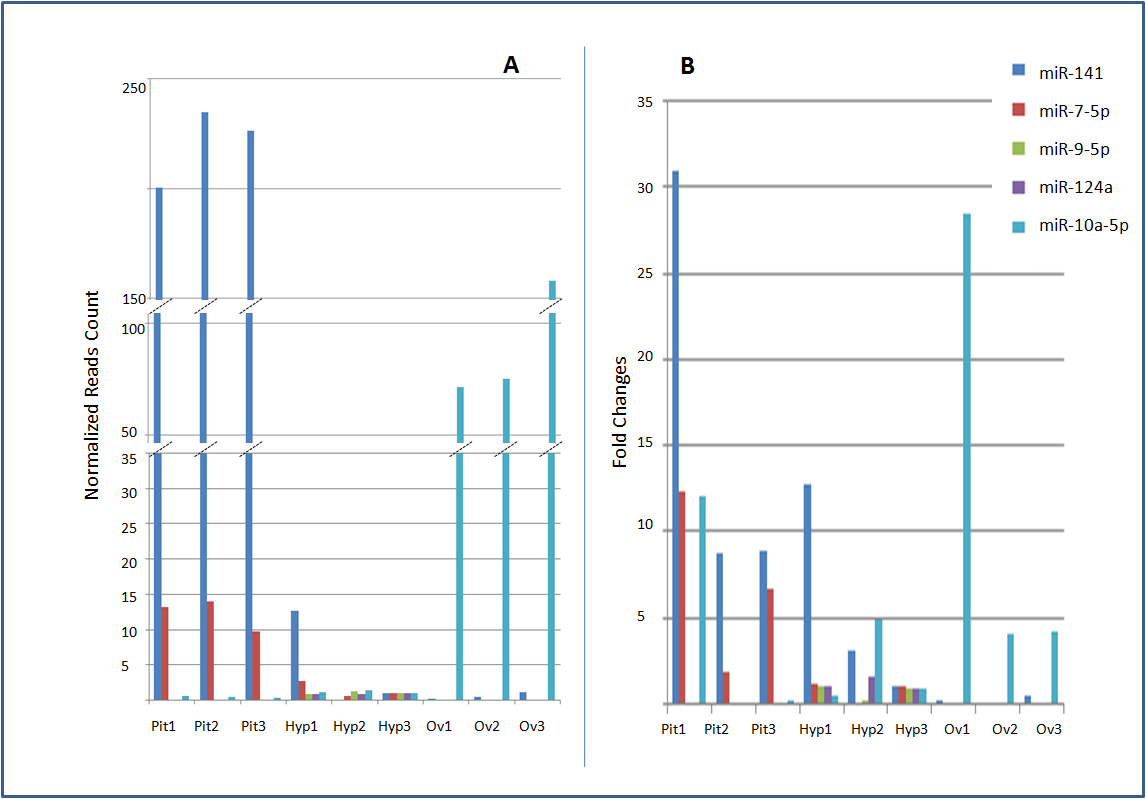


Additional File7. Comparison between A) RNA-Seq and B) Real-time PCR data, for 5 miRNAs (miR-141, miR-7-5p, miR-9-5p, miR-124a, miR-10a-5p), obtained from each organ: hypothalamus (Hyp), pituitary (Pit) and ovary (Ov) and three replicate (1, 2, 3).
